# A Metagenomic Insight Into the Hindgut Microbiota and Their Metabolites for Dairy Goats Fed Different Rumen Degradable Starch

**DOI:** 10.3389/fmicb.2021.651631

**Published:** 2021-06-07

**Authors:** Xiaoying Han, Xinjian Lei, Xuexin Yang, Jing Shen, Lixin Zheng, Chunjia Jin, Yangchun Cao, Junhu Yao

**Affiliations:** Country College of Animal Science and Technology, Northwest A&F University, Yangling, China

**Keywords:** dairy goat, GH family, Muc2, rumen degradable starch, short-chain fatty acids

## Abstract

High starch diets have been proven to increase the risk of hindgut acidosis in high-yielding dairy animals. As an effective measurement of dietary carbohydrate for ruminants, studies on rumen degradable starch (RDS) and the effects on the gut microbiota diversity of carbohydrate-active enzymes (CAZymes), and Kyoto Encyclopedia of Genes and Genomes (KEGG) Orthology functional categories are helpful to understand the mechanisms between gut microbiota and carbohydrate metabolism in dairy goats. A total of 18 lactating goats (45.8 ± 1.54 kg) were randomly divided equally into three dietary treatments with low dietary RDS concentrations of 20.52% (LRDS), medium RDS of 22.15% (MRDS), and high RDS of 24.88% (HRDS) on a DM basis for 5 weeks. Compared with the LRDS and MRDS groups, HRDS increased acetate molar proportion in the cecum. For the HRDS group, the abundance of family Ruminococcaceae and genus *Ruminococcaceae UCG-010* were significantly increased in the cecum. For the LRDS group, the butyrate molar proportion and the abundance of butyrate producer family Bacteroidale_S24-7, family Lachnospiraceae, and genus *Bacteroidale_S24-7_group* were significantly increased in the cecum. Based on the BugBase phenotypic prediction, the microbial oxidative stress tolerant and decreased potentially pathogenic in the LRDS group were increased in the cecum compared with the HRDS group. A metagenomic study on cecal bacteria revealed that dietary RDS level could affect carbohydrate metabolism by increasing the glycoside hydrolase 95 (GH95) family and cellulase enzyme (EC 3.2.1.4) in the HRDS group; increasing the GH13_20 family and isoamylase enzyme (EC 3.2.1.68) in the LRDS group. PROBIO probiotics database showed the relative gene abundance of cecal probiotics significantly decreased in the HRDS group. Furthermore, goats fed the HRDS diet had a lower protein expression of Muc2, and greater expression RNA of interleukin-1β and secretory immunoglobulin A in cecal mucosa than did goats fed the LRDS diet. Combined with the information from previous results from rumen, dietary RDS level altered the degradation position of carbohydrates in the gastrointestinal (GI) tract and increased the relative abundance of gene encoded enzymes degrading cellulose in the HRDS group in the cecum of dairy goats. This study revealed that the HRDS diet could bring disturbances to the microbial communities network containing taxa of the Lachnospiraceae and Ruminococcaceae and damage the mucus layer and inflammation in the cecum of dairy goats.

## Introduction

Due to the complex and abundant microorganisms colonized in the rumen and cecum, feed fermentation organs in ruminants are more diverse than monogastric animals (Howie and Baker, 1952; Shen et al., 2020b). Rumen has attracted a tremendous amount of attention in ruminant nutrition studies, whereas limited attention has been placed on the microorganism of the hindgut (Seshadri et al., 2018; Xue et al., 2020). Dietary carbohydrate could affect the intestinal microbiome and metabolites by affecting the digesta composition in the hindgut of human and murine (Riva et al., 2019; Deehan et al., 2020). For ruminants, high starch intake tends to decrease ruminal fiber digestibility and increase the risk of dysbiosis and the depression of fiber degradation in the hindgut (Petri et al., 2019). Previously, we found rumen degradable starch (RDS) could be an effective evaluation for ruminant dietary carbohydrate and digestive health. Increased RDS could affect cellulolytic bacteria and amylolytic bacteria in rumen and increase the potential risk for sub-acute ruminal acidosis of goats (Li et al.,2014a,b). High RDS diet decreased the cellulose and starch branching enzymes in dairy goats. Meanwhile, large amount of RDS gathered in rumen not only enhance the risk of subacute ruminal acidosis (SARA), but also cause hindgut acidosis in dairy cows (Petri et al., 2019). Increased dietary RDS represents a rich amount of undigested starch passing into the intestine. Hindgut acidosis could be characterized by accumulation of short-chain fatty acids (SCFAs), decreased pH, and damage to the intestinal-barrier such as mucin (Plaizier et al., 2018). For ruminants suffering hindgut acidosis, due to feeding on a higher proportion of grains or concentrate and lesser amounts of fiber and forage, which will increase the flow of fermentable substrate to the hindgut, undigested carbohydrate and microbial products like LPS accumulate in the hindgut (Gressley et al., 2011; Shen et al., 2020a). Those compounds could cause intestinal mucosa injury and increases expression of cytokines, such as tumor necrosis factor-α and interleukin-1β (Tao L. et al., 2017). Over 99% of the microorganisms colonizing in the intestinal tract are bacteria, which could use the undigested nutrients and non-structural CHO to supply various material for host ruminants, like energy sources, neurotransmitters, and even toxins (Steele et al., 2016; Tao S. Y. et al., 2017). Although a lot of studies used metagenomic techniques to investigate the metagenome-assembled genomes in rumen (Stewart et al., 2018, 2019), the function of microbes colonized in the intestine still did not get enough attention. At present, most research about dietary treatments in ruminants characterizing the intestinal bacterial community based on 16s rRNA sequencing analysis also have shortcomings, in that they do not include sufficient information of microbial functions (Jiao et al., 2019; Li et al., 2019). Our study is the first one combining 16S rRNA sequencing and metagenomic technologies together to evaluate the effects of dietary RDS level on intestinal bacteria, immune barrier, and carbohydrate degradation by carbohydrate-active enzymes (CAZymes) and Kyoto Encyclopedia of Gene and Genomes (KEGG) functional analysis in dairy goats. In general, this study enhanced our understanding of hindgut microorganisms in response to the changes in dietary RDS level and cellulose degradation in dairy goats.

## Materials and Methods

### Ethics Statement

In this study, all animal procedures were approved by the Institutional Animal Care and Use Committee of Northwest A&F University. The protocols of this study were specifically approved with the protocol number NWAFAC1008.

### Animals, Diets, and Experimental Design

A total of 18 lactating dairy goats with an average body weight of 45.8 ± 1.54 kg were used in this study. Two weeks before the experiment, all goats were fed *ad libitum* with a diet containing a forage-to-concentrate ratio (F: C) of 45:55 to ensure adaptation to the diet. After dietary adaptation, all goats were randomly divided equally into three dietary treatments with different RDS levels for 5 weeks. The three diets with different RDS levels were designed based on our previous study (Li et al., 2014c). All goats were fed twice per day at 0,830 and 1,630 h, and drinking water was offered *ad libitum* during the experimental period. The dietary treatments were isoenergetic, isonitrogenous, and isostarch diets with three levels of RDS: low RDS (LRDS = 20.52%), medium RDS (MRDS = 22.15%), and high RDS (HRDS = 24.88%). The calculation of RDS used the following formula: RDS = $\sum_{i=1}^{n}P\,i$ × ERD*_*i*_*, where *P_*i*_* represents the proportion of dietary starch of the feed *i* in the diet, ERD*i* represents the effective starch degradability of the feed *i*, and *n* is the number of ingredients containing starch in the feed formula. A detailed method was reported in Li et al. (2018). Corn and wheat were used to obtain the required RDS level. The dietary composition and nutrient contents of the experimental diets are presented in Supplementary Table 1.

### Sample Collection

After a 5-week feeding period, all goats were slaughtered 3 h after morning feeding under combined anesthesia {intramuscular injection of xylazole and dihydroetorphine hydrochloride [1.5 mL (100 kg body weight); Lumianning; HUAMU, Jilin, China]}. After death, the intestine (jejunum, cecum, colon, and rectum) were separated and the digesta of jejunum, cecum, and rectum were aseptically collected and frozen (frozen by liquid nitrogen in the first step then stored at −80°C) for further analysis. The intestinal mucous of the cecum was separated from the muscular layers and carefully harvested. The intestine tissues of the cecum were collected in 2 × 2 cm^2^ pieces and fixed in 4% paraformaldehyde to make paraffin-embedded tissues. The pH of the digesta in the jejunum and cecum were measured by mobile pH meter (Ohaus Instruments Co., Ltd., China).

### Determination of Intestinal Digesta Composition

The cellulose content in the cecum was determined by Cellulose Content Kit (Nanjing Jiancheng Bioengineering Institute CO., Ltd., China). The amylose content in the cecum was determined by Megazyme Total Starch Kit (K-TSTA 04/2009, Biostest Co., Ltd., China). The acetate, propionate, butyrate, valerate, isobutyrate, and isovalerate concentrations of hindgut digesta were determined by Agilent 7820A GC system (Agilent, CA, United States) (Li et al., 2014b). About 0.5 g digesta was weighed and diluted with 1 mL ultrapure water. Then, a high-speed homogenizer Scientz-48 (Ningbo Scientz Bioscience Co., Inc., China) was used to make sure the combination was mixed completely. After being centrifuged by centrifugal 5810R (Eppendorf, United States) at 13,500 rpm about 10 min, 1.5 mL supernatant of diluted digesta was moved into 300 μL 25%w/v metaphosphoric solution for further purification. After repeating the previous centrifugation step, 1 mL supernatant was moved into 200 μL 25% crotonic acid, and the mixture was collected into an EP tube passed through a 0.5 μm filter to be measured.

### Bacterial DNA Extraction and PCR Amplification

The total microbial DNA was extracted from intestinal digesta using the E.Z.N.A.^®^ soil DNA Kit (Omega Bio-tek, Norcross, GA, United States.). The concentrations of all extracted nucleic acid samples were detected by spectrophotometry Nanodrop 2000 (Thermo Fisher Scientific, Wilmington, DE, United States), and purity was monitored with 1% agarose gels. All extracted DNA samples were stored at −80°C for further analysis. The amplicon library preparation was performed by polymerase chain reaction amplification of the V3-V4 region of the 16S rRNA gene using the primer pairs: 338F (5′-ACTCCTACGGGAGGCAGCAG-3′) and 806R (5′-GGACTACHVGGGTWTCT-AAT-3′) by thermocycler polymerase chain reaction system ABI GeneAmp 9700 (Thermo Fisher Scientific, Wilmington, DE, United States) The polymerase chain reactions amplification refer to the previous study (Zhang et al., 2020).

### 16S rRNA Sequencing Analysis

After library construction, the samples were sequenced on an Illumina MiSeq platform using HiSeq 3000/4000 PE Cluster Kit and HiSeq 3000/4000 SBS Kit (Altschul et al., 1997). The raw reads were demultiplexed, quality-filtered, and analyzed by Quantitative Insight Into Microbial Ecology software [refer to previous study by Navas-Molina et al. (2013)]. The sequences were clustered into operational taxonomic units (OTUs) at a 97% similarity threshold using UPARSE (version 7.1^1^) (Edgar et al., 2011). Silva v132 Database based on the mothur version 1.30.1 algorithm was used to select, annotate, and normalize OTUs (Schloss et al., 2009; Glöckner et al., 2017). Sequences were submitted to the NCBI short read archive (SRA) under accession numbers SRP295458.

The principle coordinate analysis (PCoA) graphs were enabled the between-samples comparison (beta diversity) of the microbial communities. The PCoA of weighted UniFrac matrices with statistical significance was determined by Permutational Multivariate Analysis of Variance (PERMANOVA) (Lozupone et al., 2011; Dong et al., 2019). The resulting OTUs were submitted to BugBase^2^ which could calculate differences between groups in terms of microbial phenotypes in the cecum: microbial oxidative stress tolerant and potentially pathogenic (Thomas et al., 2016). Differential abundance of family, genus, and predicted microbial phenotypes was tested by SPSS software (version 20.0) using the one-way ANOVA model across the three treatments.

### Metagenome Library Preparation and Sequencing

Digesta DNA from the jejunum, caecum, and rectum were fragmented to obtain fragments of an average size of 400 bp, using Covaris M220 (Gene Company Limited, China) for paired-end library construction. Paired-end library preparations were performed according to the NEXTFLEX Rapid DNA-Seq (Bioo Scientific, Austin, TX, United States). Adapters containing the full complement of sequencing primer hybridization sites were ligated to the blunt end of fragments. After library construction, the paired-end sequencing was performed on Illumina HiSeq4000 platform (Illumina Inc., San Diego, CA, United States) located at Majorbio Bio-Pharm Technology Co., Ltd. (Shanghai, China) using HiSeq 3000/4000 PE Cluster Kit and HiSeq 3000/4000 SBS Kit according to the manufacturer’s instructions^3^.

### Sequence Quality Control and Genome Assembly

The data were analyzed on the free online platform of Majorbio Cloud Platform^4^. SeqPrep (Verision 1.1) was used to strip the adapter sequence from the 3′ and 5′ end of paired-end Illumina reads. The paired-end Illumina reads were trimmed of adaptors, and low-quality reads (shorter than 50 bp or with an average quality score < 20 or having N bases) were removed by Sickle (Verision 1.33). Then, host reads were filtered by aligning reads against Capra hircus genome with BWA (Version 0.7.9a) and removing reads with high scoring alignments host. The Megahit^5^ (version 1.1.2), which makes use of SdBG was used to assemble different depth sequences and get the statistical table of gene prediction results. Contigs with lengths of 300 bp or over were selected as the final assembling result, and then the contigs were used for further gene prediction and annotation. Sequences were submitted to the NCBI short read archive (SRA) under accession numbers PRJNA706869.

### Gene Prediction, Taxonomy, and Functional Annotation

We used Metagene to predict the Open reading frames (ORFs) from each assembled contig (Noguchi et al., 2006). Then, we used the NCBI translation table to translate predicted ORFs over a length of ≥ 100 bp into amino acid sequences. Then we clustered the database with CD-HIT software^6^ (default parameters: 95% identity, 90% coverage) and took the longest gene of each class as a representative sequence (Fu et al., 2012). A non-redundant gene set was constructed and compared with the optimized reads (default parameter: 95% identity). The abundance information of genes was counted by Soapaligner software, and evaluated *via* reads per kilobase per million mapped (RPKM) in each sample. The taxonomic information was annotated by a BLASTp (Version 2.2.28 +) search in the NCBI NR database [refer to previous study by Altschul et al. (1997)]; the KEGG annotation was conducted by KOBAS 2.0 against the KEGG database (Version: 2018-07-30); the carbohydrate active enzymes annotation was conducted by hmmscan^7^ against CAZymes database Version 6.0. All these databases had an E-value cutoff of 1E-5 while annotating ORFs.

### Quantitative Real-Time Quantitative PCR

The expression levels of six genes (*IL1B*, *IL 12*, *IkB*, *NF-kB*, and *IFN-*γ) associated with mucosal immunity were analyzed by RT-qPCR. The total RNA was extracted by the Trizol (Takara, Dalian, China) method from intestinal mucosa samples and reverse transcribed by the Prime Script^®^ RT reagent kit (Takara, Dalian, China) (Chang et al., 2015). The housekeeping gene GAPDH was used as an internal normalization control (Vandesompele et al., 2002). All samples were examined in triplicate and analyzed using the 2^–ΔΔ^ ^Ct^ method (Livak and Schmittgen, 2001).

### Immunohistochemistry

Immunohistochemistry was performed [refer to the previous study by Dong et al. (2015)]. The cecum was removed and fixed in 4% paraformaldehyde/PBS overnight at 4°C. The tissue blocks were dehydrated and embedded in paraffin. The 5 μm thick serial sections were cut in a coronal plane under a Leica microtome (Leica 2016, Germany) and mounted on 0.1% polylysine reagent (Sigma, Germany) coated slides. Then, rehydrated and ethanol cleared sections were incubated with 3% H_2_O_2_-methanol (ZLI-9064, ZSGB-BIO, Beijing, China) for 10 min at room temperature, and washed in PBS for 3 min × 5 min. Slides were treated with microwaves (700 W) in 0.01 mol/L citrate-buffered saline (pH 6.0) for 2 min × 5 min for antigen retrieval and washed in PBS for 2 min × 5 min. Then, the sections were incubated in 10% normal goat serum (ZLI-9021, ZSGB-BIO, Beijing, China) in PBS for 20 min. Sections were next incubated with rabbit anti-Muc2 (1:50, GB11344,Servicebio, Wuhan, China) overnight at 4°C. Thereafter, they were exposed to biotinylated second antibody biotinylated goat anti-rabbit IgG (SP-9001, ZSGB-BIO, China) for 30 min at 37°C. Color development was performed using a diaminobenzidine (DAB) kit (K135925C, ZSGB-BIO, Beijing, China) according to the manufacturer’s instructions. Images were digitized using a microscope (BA400Digital, Motic, Xiamen, China) and analyzed using Image-Pro Plus 6.0 software (Media Cybernetics, Silver Spring, Maryland, United States).

### Enzyme-Linked Immunosorbent Assay in Cecum

Enzyme-Linked Immunosorbent Assay (ELISA) kits were employed to measure the concentrations of CD4^+^ T Cells, CD8^+^ T Cells (Shanghai Mlbio Institute Co., Ltd, China), and SIgA (Nanjing Jiancheng Bioengineering Institute Co., Ltd, China), following the manufacturer’s instructions. The absorbance of each solution was determined at a wavelength of 450 nm (Colón-Díaz et al., 2020).

### Statistical Analysis

Statistical comparisons based on statistical software package (SPSS version20.0; United States) with a false discovery rate (FDR) value < 0.05 to correct the *P* values. Differential abundance of bacteria on family and genus level (jejunum, cecum, and rectum), CAZymes and KEGG enzymes (cecum) were tested by one-way ANOVA among three groups, Student’s *t* test was used between two groups, omics data was tested using the SPSS software (version20.0; Chicago, IL, United States), and the significance was defined by Duncan’s test among more than two groups. Taxonomic data in jejunum, cecum and rectum were analyzed on the online platform of Majorbio Cloud Platform^8^, and so did the functional data in cecum. The Differences were statistically significant at *P* < 0.05 or a tendency of difference at 0.05 ≤ *P* < 0.10. Spearman’s rank correlation coefficients were used to examine the correlations between bacterial abundance and SCFAs concentrations.

## Results

### Carbohydrates and SCFAs Composition in Hindgut

The concentration of total SCFAs and molar proportions of individual SCFAs were shown in Table 1. There was an increased tendency in the concentrations of total SCFAs in the LRDS group than the HRDS group at the cecum and colon (0.05 ≤ *P* < 0.10), and the molar proportion of various SCFAs were compared. The results showed that butyrate was decreased in the HRDS group at the colon and rectum (*P* < 0.05) compared with the MRDS group; acetate was increased in the HRDS group (*P* < 0.05) at each position of the hindgut (cecum, colon, and rectum) compared with the LRDS group. To quantify the contribution between the molar proportion of butyrate and acetate, we used butyrate: acetate (B:A) ratio in this study. Compared with the LRDS group, the B:A ratio was decreased in the HRDS group at the colon (*P* < 0.05), rectum (*P* < 0.05), and had a downward trend at the cecum (0.05 ≤ *P* < 0.10). We also found that the molar proportion of propionate was higher in the MRDS group (*P* < 0.05) compared with the HRDS group. There was no difference in pH between different RDS groups (Table 2; *P* > 0.05). The starch and cellulose are also analyzed in intestinal digesta. As Supplementary Figure 1 shows, the content of cellulose in the HRDS group was higher (*P* < 0.05) than the MRDS group. The determination of amylose content showed that cecum digesta in the HRDS group had a lower amylose content (*P* < 0.05) compared with the LRDS group.

**TABLE 1:** Effect of dietary RDS on hindgut SCFAs concentration.

Item	Treatments^1^			SEM^2^	*P*-value
	LRDS	MRDS	HRDS		
**A**					
Total SCFAs, mM	29.39	24.93	21.75	1.377	0.07
**SCFAs proportion, mol/100 mol**					
Acetate (A)	66.35^b^	67.05^b^	71.42^a^	0.013	0.05
Propionate	16.92	17.76	16.25	0.007	0.38
Isobutyrate	0.72^b^	0.68^b^	1.15^a^	0.001	0.01
Butyrate (B)	14.05	12.33	8.40	0.016	0.08
Isovalerate	0.58^b^	0.49^b^	0.99^a^	0.001	0.01
Valerate	1.38	1.69	1.79	0.001	0.15
B:A ratio	0.22	0.19	0.12	0.003	0.10
**B**					
Total SCFAs, mM	27.65	24.86	21.95	1.051	0.08
**SCFAs proportion, mol/100 mol**					
Acetate (A)	62.02^b^	62.53^b^	68.09^a^	0.017	0.05
Propionate	16.16	18.07	15.76	0.011	0.30
Isobutyrate	1.04	0.86	1.23	0.001	0.20
Butyrate (B)	18.54^a^	16.33^ab^	12.57^b^	0.013	0.03
Isovalerate	0.96	0.88	0.89	0.001	0.85
Valerate	1.28	1.34	1.46	0.002	0.73
B:A ratio	0.30^a^	0.27^ab^	0.19^b^	0.003	0.03
**C**					
Total SCFAs, mM	23.93	23.36	23.10	1.170	0.96
**SCFAs^3^ proportion, mol/100 mol**					
Acetate (A)	61.92^b^	61.97^b^	69.23^a^	0.013	< 0.01
Propionate	15.34^ab^	18.18^a^	14.79^b^	0.009	0.05
Isobutyrate	1.15	1.03	1.14	0.001	0.51
Butyrate (B)	18.99^a^	16.20^a^	11.19^b^	0.016	0.02
Isovalerate	1.02	0.98	1.09	0.001	0.69
Valerate	1.58	1.65	1.60	0.001	0.94
B:A^4^ ratio	0.31^a^	0.27^a^	0.16^b^	0.003	0.01

*(A), cecum; (B), colon; (C), rectum.*

*^*a–c*^Means within the same row with different superscripts differ significantly (*P* < 0.05).*

*^1^Treatments were the LRDS diet (RDS = 20.52%), MRDS diet (RDS = 22.15%), and HRDS diet (RDS = 24.88%) with similar total starch contents.*

*^2^SEM = Standard Error of Mean.*

*^3^SCFAs = short chain fatty acids.*

*^4^B:A = butyrate: acetate.*

**TABLE 2:** Effect of dietary RDS on intestinal pH level.

Item	Treatments^1^			SEM^2^	*P*-value
	LRDS	MRDS	HRDS		
Jejunum	6.63	6.96	6.81	0.144	0.68
Cecum	6.86	7.15	7.29	0.117	0.33

*^*a–c*^Means within the same row with different superscripts differ significantly (*P* < 0.05).*

*^1^Treatments were the LRDS diet (RDS = 20.52%), MRDS diet (RDS = 22.15%), and HRDS diet (RDS = 24.88%) with similar total starch contents.*

*^2^SEM = Standard Error of Mean.*

### High RDS Diet Induced Shift in SCFAs-Producing Community and Cellulolytic

**FIGURE 1:**
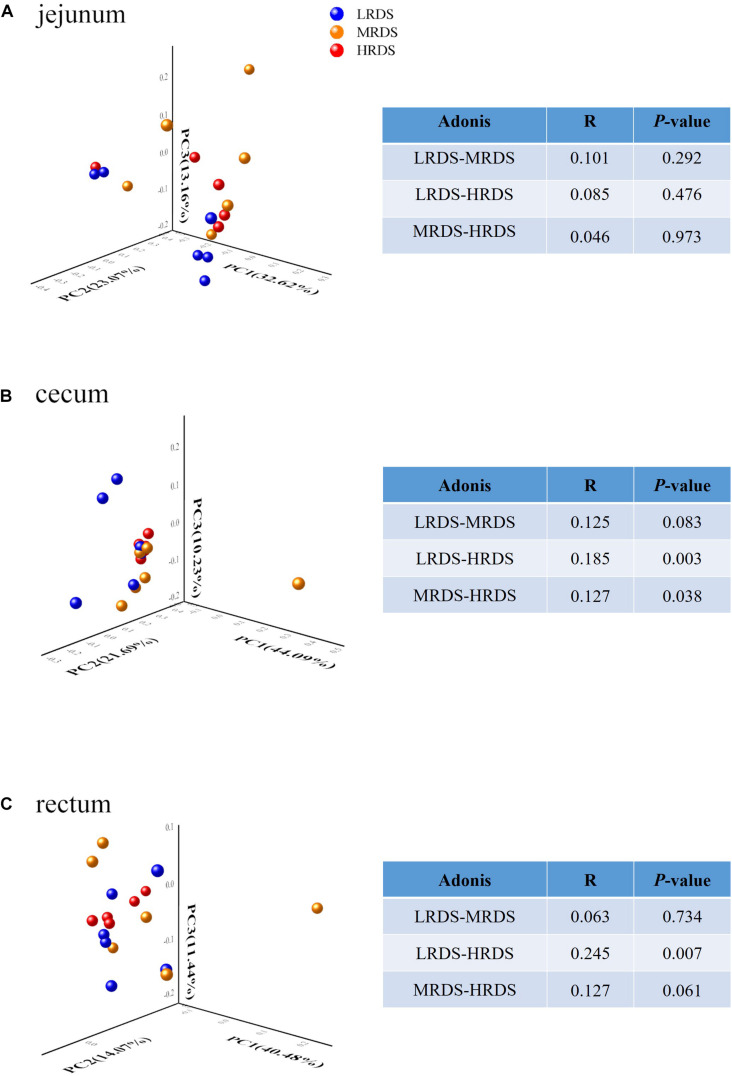
Microbial community difference among different groups in the jejunum microbiome **(A)**, but showing obvious dysbiosis over positions in the cecum **(B)** and rectum microbiome **(C)**. The data represent the comparison of the gut bacterial profiles among the LRDS (blue), MRDS (yellow), and HRDS (red) groups. Principal coordinate analysis and Adonis scores computed for OTU taxon illustrating the grouping patterns based on the unweighted UniFrac distances. Distances between each pair of samples represent their dissimilarities. The R value represents the effect size indicating that the percent variation can be explained by RDS level. Only taxa meeting an LDA significant threshold > 2 are shown.

The comparisons of microbial beta-diversity based on PCoA analysis and Adonis test were given in Figure 1. The LRDS, HRDS, and MRDS groups in the jejunum cannot be distinguished. Moreover, the HRDS group can be distinguished from LRDS (*P* < 0.05) and MRDS (*P* < 0.05) in the cecum. In the rectum, the HRDS group can be distinguished from LRDS (*P* < 0.05) and had a trend of separation with MRDS (0.05 ≤ *P* < 0.1).

**FIGURE 2:**
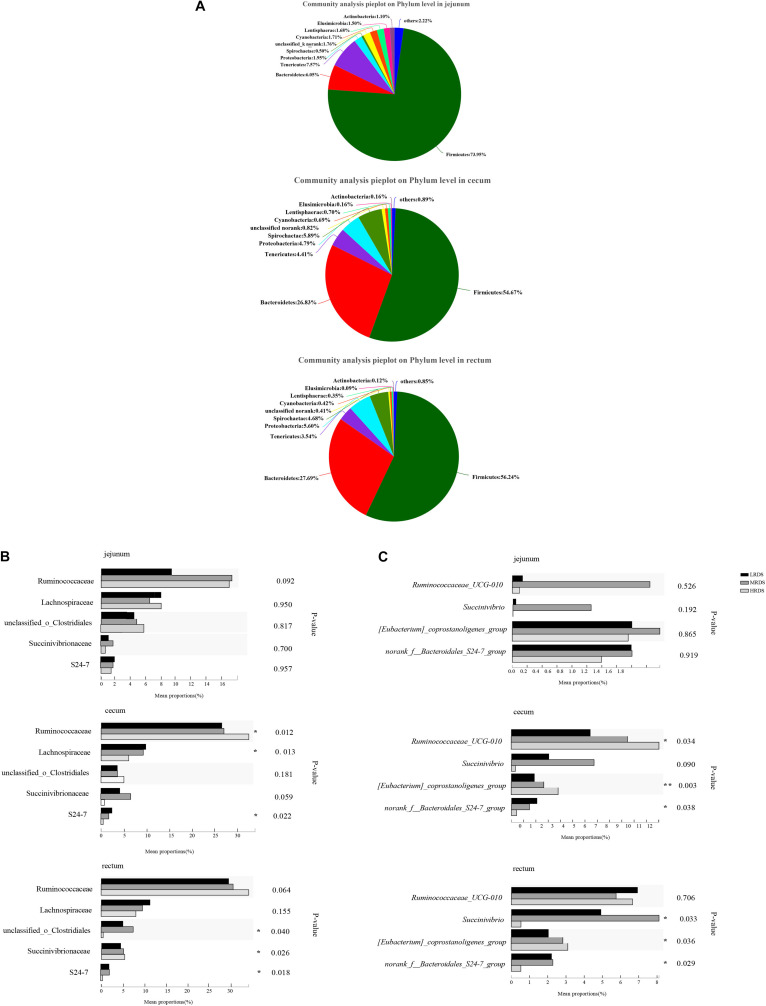
Bacterial community composition in each intestinal tract region **(A)** on phyla level, **(B)** family level and, **(C)** genus level. **(B)** Comparison of relative abundances of the five main bacterial families (Ruminococcaceae, Lachnospiraceae, O. Clostridiales, S24-7, and Succinivibrion- aceae) among groups throughout the GIT region. **(C)** Comparison of relative abundances of the four main bacterial genera (*Ruminococcaceae UCG-010*, *Eubacterium_coprostanoligenes, Bacteroidale_S24-7*_*group*, and *Succinivibrio*) among groups throughout the intestinal tract region. Asterisk (*) denotes significant correlations between hind-gut microbiome abundance with parameters *P < 0.05, **P < 0.01.

To illustrate whether the changes of SCFAs were associated with the acid-produced bacteria, we compared the number of significantly changed bacteria based on 16s rRNA sequencing (Figure 2). A total of 22 phyla, 130 families, and 214 bacterial genera were identified in all samples, including the five most abundant phyla: Firmicutes, Bacteroidetes, Spirochetes, Tenericutes, and Proteobacteria (Figure 2A). To identify the specific bacterial taxa affected by RDS, we compared the families and genera based on one-way ANOVA analysis (Figures 2B,C). The results revealed the most abundant family was classified as Ruminococcaceae and the most abundant genus was classified as F_Ruminococcaceae. On a family level, compared to the LRDS group, HRDS increased the relative abundance *of* Ruminococcaceae (*P* < 0.05), decreased the relative abundanc*e of* Lachnospiraceae *and Bacteroidales_S24-7* (*P* < 0.05) in samples collected from the cecum, and also increased the relative abundance of Bacteroidales_S24-7 (*P* < 0.05) in the rectum. MRDS increased the relative abundance of Succinivibrionaceae (*P* < 0.05) compared to the LRDS group in the cecum, and increased the relative abundance of Succinivibrionaceae (*P* < 0.05) compared to the HRDS group in rectum (Figure 2B).

On a genus level, *Ruminococcaceae UCG-010* was the most abundant with an increase in the HRDS group at the cecum (*P* < 0.05) compared with the LRDS group (Figure 2C). The HRDS group increased the relative abundance of *Eubacterium_coprostanoligenes_group* (*P* < 0.05) and decreased the relative abundance of *Bacteroidales_S24-7_group_unidentified* (*P* < 0.05) in the rectum and cecum. The MRDS group increased the relative abundance of *Succinivibrio* in the rectum (*P* < 0.05) and had an uptrend in the cecum (0.05 ≤ *P* < 0.10) compared with the HRDS group (Figure 2C). In the jejunum, there was no significant change (*P* > 0.05) in previous genera or 15% top abundance, reflecting that dietary RDS played an even more important role in the composition of bacterial in hindgut than the small intestine.

**FIGURE 3:**
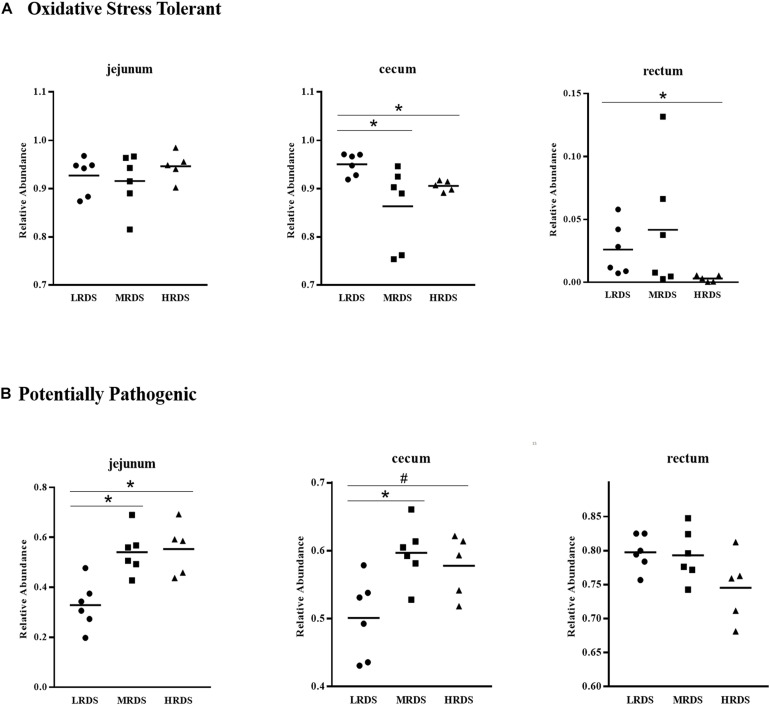
BugBase phenotypes prediction. **(A)** The oxidative stress tolerant. **(B)** The potentially pathogenic *P < 0.05, ^#^0.05 < *P* < 0.10.

The BugBase phenotypes prediction was presented in Figure 3. The results show that the LRDS group increased the microbial oxidative stress tolerant phenotypes in the cecum (*P* < 0.05) compared with the MRDS and HRDS groups, as well as in the rectum (*P* < 0.05) compared to the MRDS group. Compared to the MRDS and HRDS groups, the potentially pathogenic phenotypes of the LRDS group were reduced (*P* < 0.05) in jejunum as well as in the cecum (0.05 ≤ *P* < 0.10).

**FIGURE 4:**
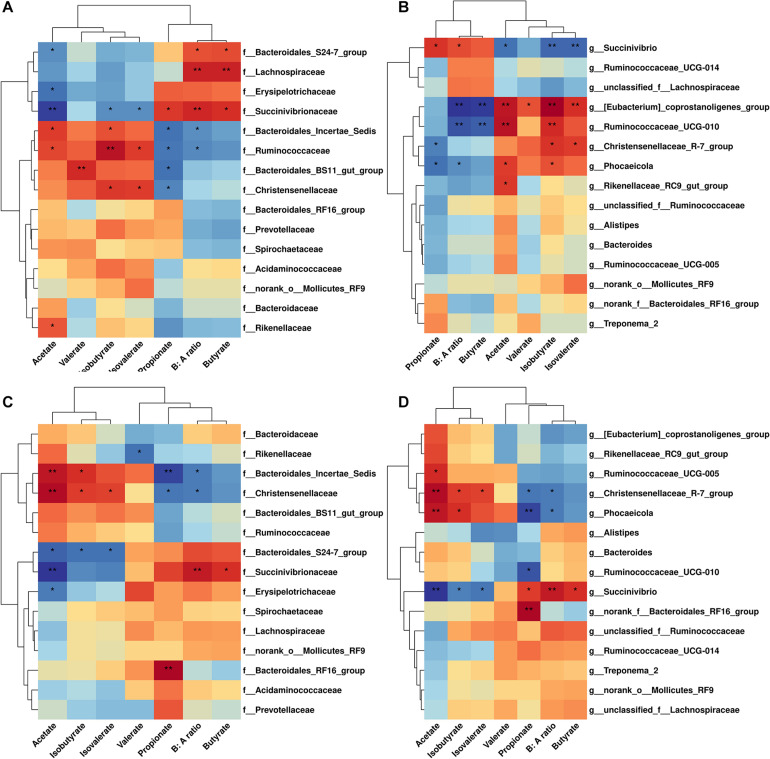
Correlation analysis between microbiome abundance and SCFAs concentration in cecum and rectum.**(A)** Family level in cecum. **(B)** Genus level in cecum. **(C)** Family level in rectum. **(D)** Genus level in rectum. Cells are colored based on spearman correlation coefficient. Only relative abundance of the 15 most-abundant bacterial families or genera are shown.

Correlation analysis of cecal microbiome abundance with microbial metabolites were shown in Figure 4. There were nine families related with SCFAs molar proportion (*P* < 0.05) (Figure 4A). The strongest positive correlation with the molar proportion of acetate was found in Bacteroidale_incertae_sedis, Rikenellaceae, and Ruminococcaceae; the strongest negative correlation with the molar proportion of acetate was found in Bacteroidale_S24-7, Erysipelotrichaceae, and Succinivibrionaceae. In addition, Lachnospiraceae and Succinivibrionaceae were positively related with butyrate molar proportion and B: A ratio. There were six genera significantly related with SCFAs molar proportion (*P* < 0.05; Figure 4B). Besides the genera *Ruminococcaceae_UCG-010* and *Rikenellaceae_RC9_gut_group* we mentioned previously, the *Eubacterium_coprostanoligenes group* and *Phocaeicola* were also positively correlated (*P* < 0.05) with the molar proportion of acetate. Genus *Succinivibrio* was negatively correlated with the acetate molar proportion, while positively correlated with the molar proportion of butyrate, propionate, and B: A ratio. Genus *Eubacterium_coprostano-ligenes_group* and *Ruminococcaceae_UCG-010* were negatively correlated with butyrate molar proportion and B: A ratio.

### Metagenomic Analysis Found That High RDS Diet Disturbed Microbial Function by Affecting Genes Encoding Carbohydrate Enzymes

**FIGURE 5:**
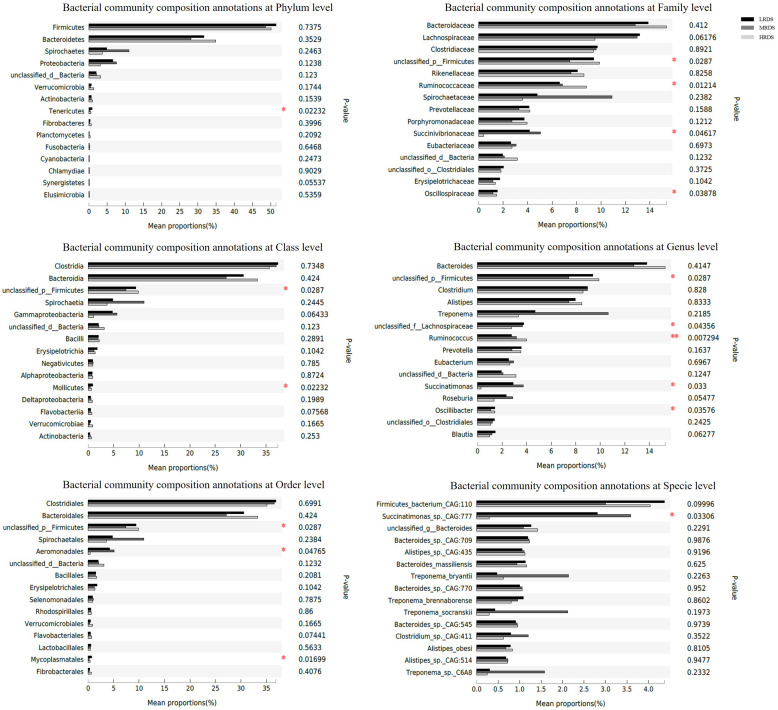
Relative gene abundance of microbial metagenomics compared with NR database in cecum. On phylum, class, order, family, genus, and species levels.

To gain insight into the molecular functions of gut microbiota, metagenomics analysis was used to analyze the contribution of microbial function in the cecum. Using BLASTp to compare non-redundant gene sets based on the NR database, we compared the relative abundance of microbial genes among groups. These results were shown in Figure 5. Of the 78 identified phyla, Firmicutes was predominant in cecum samples. On species level, *Succinatimonas_sp._CAG:777* was the second most abundant in the cecum, and was increased in the MRDS group compared with the LRDS group (*P* < 0.05).

Compared to the LRDS and MRDS groups, the HRDS group decreased (*P* < 0.05) the relative abundance of class Mollicutes, order Mycoplasmatales, and phylum Tenericutes. Compared with the HRDS group, the MRDS group decreased (*P* < 0.05) the relative abundance of *unclassified_p__Firmicutes*. In addition, the MRDS group increased the relative abundance of the family Succinivibrionaceae (*P* < 0.05) and genus *Succinatimonas* compared to the HRDS group.

**FIGURE 6:**
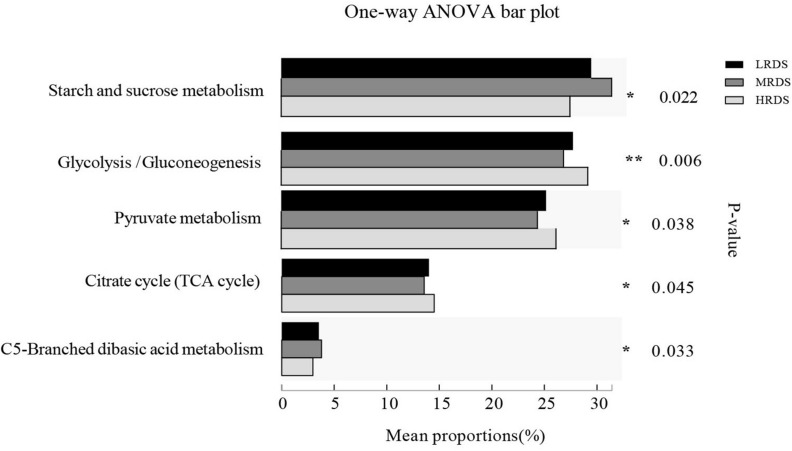
KEGG pathways corresponded to carbohydrate metabolism with significant changes.

**FIGURE 7:**
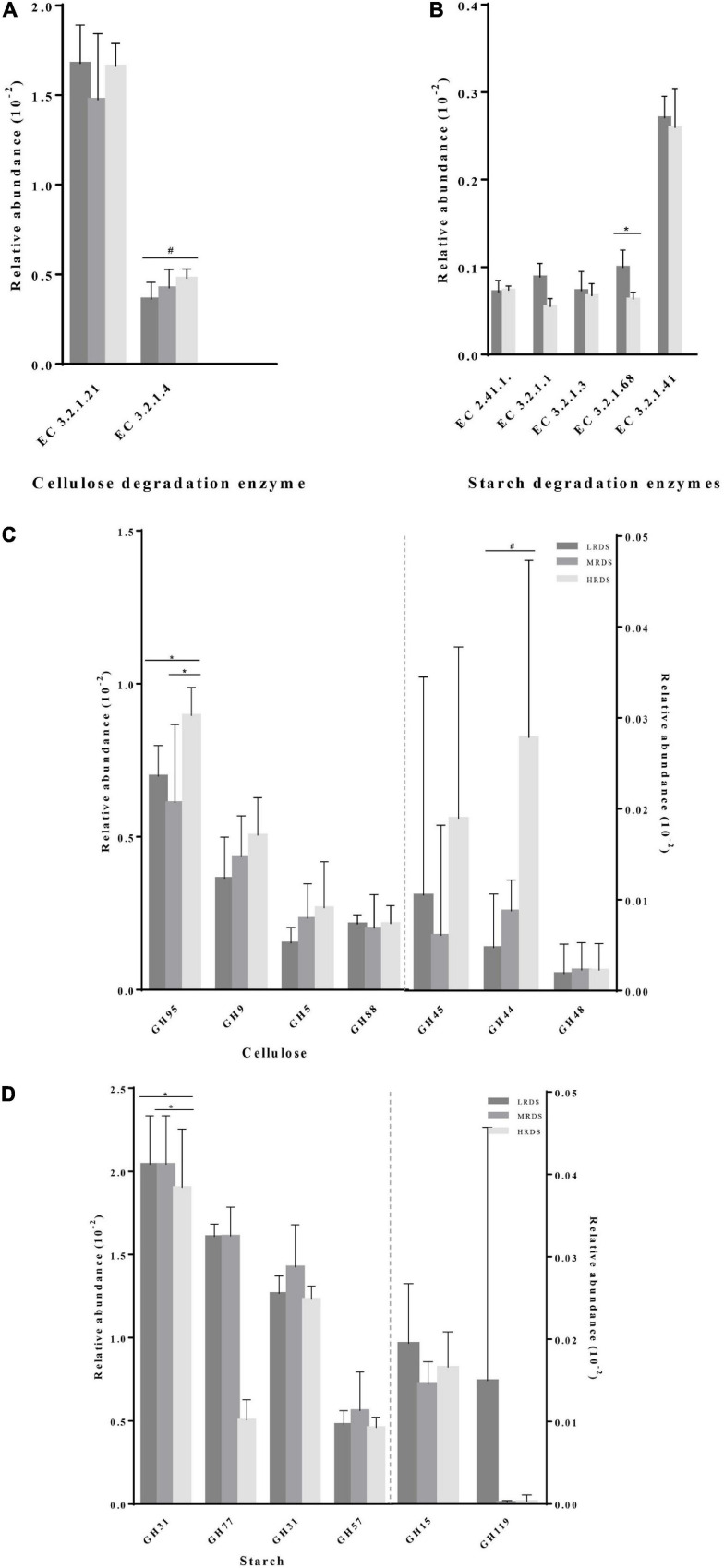
Comparisons of the gene abundance of KEGG enzymes and GH family for **(A,C)** cellulase and **(B,D)** amylase.

Comparison of the functional capacity of the microbiota could help us investigating the metabolic differences among groups. In this study, we used Gene set enrichment analysis (GSEA) to identify differentially abundant KEGG pathways. Finally, we identified 15 KEGG pathways in carbohydrate metabolism at level 3, five of them changed in mean proportion among groups (Figure 6), including starch and sucrose metabolism, Glycolysis/Gluconeogenesis, Pyruvate metabolism, Citrate cycle (TCA cycle), and C5-Branched dibasic acid metabolism (*P* < 0.05). The KEGG database showed that there were various enzymes encoded by corresponding functional genes which took part in carbohydrate metabolism in the cecum, including cellulase (endoglucanases, glucosidase, and glycosylase) and amylase (α-amylase, glycogen phosphorylase, and glucosidase) (Figures 7A,B). Within the process of cellulose degradation, the genes encoding cellulase enzyme (EC 3.2.1.4) were increased (0.05 ≤ *P* < 0.10) in the HRDS group compared with the LRDS group. The abundance of amylopectin-degrading enzyme isoamylase (EC 3.2.1.68) was lower (*P* < 0.05) in the HRDS group compared with the LRDS group.

We also investigated the functional information of genes annotated by the CAZymes database, and the results corresponded with KEGG annotation. When comparing the gene abundance of the carbohydrate-active enzymes family, we found that there were seven GH families (GH95, GH9, GH5, GH88, GH45, GH44, and GH48) mainly found to be associated with cellulolytic functions. The abundance of GH95 was increased in the HRDS group (*P* < 0.05) compared with the LRDS group. Six GH families (GH13_20, GH77, GH31, GH57, GH15, and GH19) were found to be mainly associated with starch degradation, and the abundance of GH13_20 was decreased in the MRDS and LRDS groups (*P* < 0.05) compared with the HRDS group (Figures 7C,D).

**FIGURE 8:**
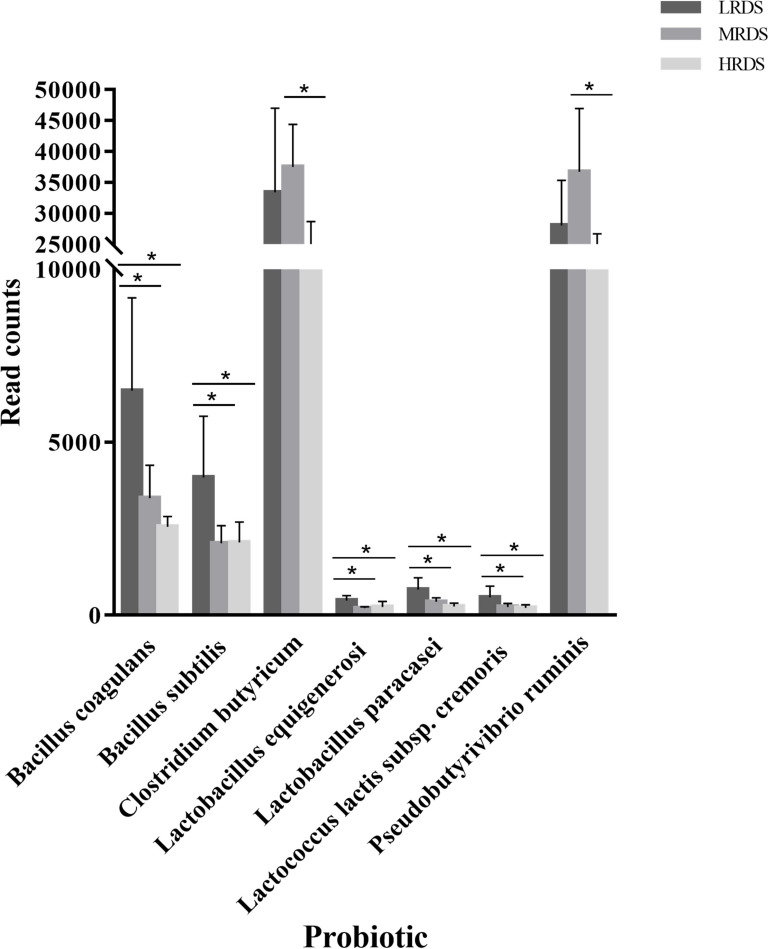
Comparisons of the abundance of the Probio annotated probiotics.

We further analyzed the genes differentially expressed among groups by the Probio database, a database of probiotics functions and lineages (Figure 8). We found that compared to the MRDS and HRDS groups, the LRDS group had an increased (*P* < 0.05) gene abundance of *Bacillus coagulans*, *Bacillus subtilis*, *Lactobacillus equigenerosi*, *Lactobacillus paracasei*, and *Lactococcus lactis* ssp. *Cremoris*. Compared with the MRDS and HRDS groups, *Clostridium butyricum* and *Pseudobutyrivibrio ruminis* were enriched (*P* < 0.05) in the MRDS group and decreased (*P* < 0.05) in the HRDS group.

### High RDS Diet Upregulated Healthy Risk by Increasing Intestinal Inflammation

**FIGURE 9:**
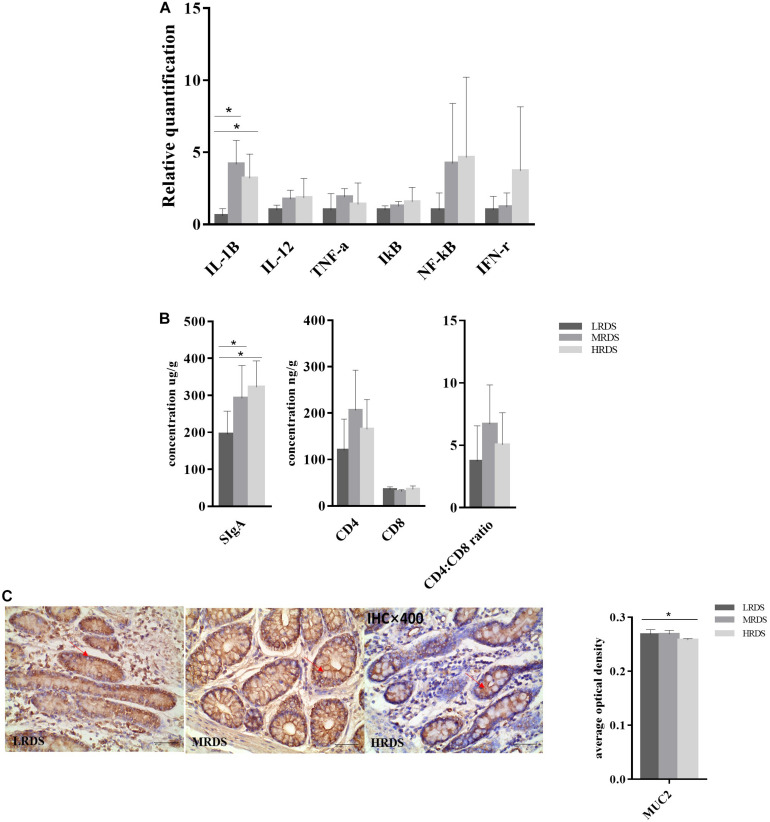
Intestinal mucosal immune in cecum. **(A)** Gene expression in the cecal mucosa. Glyeraldehyde 3-phosphate dehydrogenase (GAPDH) was used as the reference gene for gene expression including *IL-1*β, *IL-12, I*κ*B, NF-*κ*B, TNF-*α, and *IFN-*γ. **(B)** Antigen specific proteins expression of *SIgA*, CD4^+^ T cell, CD8^+^ T cell, and CD4^+^: CD8^+^ ratio in the cecal mucosa. **(C)** Antigen specific proteins expression of Muc2 and tissue localization of Muc2 in the cecum. The goblet cells contain Muc2 (brown), and mucin stains are shown in serial sections on the same tissue area.

We found that the relative expression of genes *IL-1*β (immunoregulatory cytokines interleukin 1 beta), *IL-12* (interleukin 12), *TNF-*α (tumor necrosis factor alpha), IκB (inhibitor of nuclear factor kappa B kinase), *NF*κ*-B* (nuclear factor kappa B subunit), and *IFN-*γ (interferon gamma) were detectable in the cecum mucosa. The mRNA expression of *IL-1*β (*P* < 0.05) was decreased in the LRDS and MRDS groups (Figure 9A). We found that the MRDS and HRDS groups had a significant difference of relative concentration of secretory immunoglobulin A (SIgA) in cecum mucosa tissues compared with the MRDS and HRDS groups (Figure 9B). Dietary RDS did not influence (*P* > 0.05) the relative protein concentration of CD4^+^ T Cells, CD8^+^ T Cells, and CD4^+^: CD8^+^ ratio. As Figure 9C shows, Muc2 average optical density in crypt goblet cells of the HRDS group was decreased compared with the MRDS group (*P* < 0.05).

## Discussion

We have found that RDS can be used as an effective measurement of dietary carbohydrate and digestive health in ruminants (Shen et al., 2020b). As the RDS level increased, more starch would be degraded in rumen and less starch would reach the intestine when the three treatment diets were formulated to be an isostarch (Li et al., 2014c).

It is generally known that large amounts of starch are rapidly degraded in the rumen when SARA happens (Plaizier et al., 2017). Some investigations indicated that damage to the hindgut could also occur in association with ruminal acidosis (Petri et al., 2019). In a previous study, we observed that the HRDS diet induced SARA in dairy goats (Zheng et al., 2020). However, no difference in pH level was found in any segments of intestine we detected, and no animals had diarrhea or other digestive problems, suggesting that rumen acid accumulation and inflammation induced by systemic entry of endotoxins did not cause observable hindgut acidosis in the HRDS group. Our previous study shown that HRDS altered the compositions of volatile fatty acid (VFAs) in rumen (Shen et al., 2020b). For ruminants, the fermentation site (rumen and hindgut) producing VFAs is more diverse than monogastric animals (Jin et al., 2018). The VFAs produced in the cecum account for 12% of the total VFA production due to approximately 17% cellulose degradation occurring in the cecum (Faichney, 1968). We hypothesized that a high cecal cellulose concentration in the HRDS group was one of the effects of SARA, which may explain the reduced cellulose digestive rate in rumen. However, we cannot prove conclusively why the MRDS group seems to have had a higher concentration of cellulose compared with the LRDS group, and the content may cause the high abundance of Succinivibrionaceae and *Succinivibrio* which could promote the digestion of cellulose and high propionate concentration in the hindgut.

Amylose in corn is recognized to be slowly digested in rumen because the linear structure and proteins wrapper allow it to be extensively passed into the hindgut (Larson and Hoffman, 2008). In our experiment, the increased cecal amylose concentration in LRDS-fed goats is speculated to be related to the more uniform distribution at each site of the gastrointestinal tract (GIT). Previous studies show that high amylose increased cecal SCFAs concentrations in rats and piglets (Nagata et al., 2019; Gao et al., 2020). Our study further verified that feeding diets with different RDS levels could alter the hindgut digesta composition of dairy goats thereby influencing the synthesis of SCFAs. Combined with the previous studies, we assumed that the SCFAs profile can be altered by the concentration of cellulose and amylose among groups (Matt et al., 2018; Kumar et al., 2020).

Gut microbiota plays an intermediary role between carbohydrate digestion and SCFAs production (Deehan et al., 2020). In the hindgut, microbial fermentation take over most assignments of digesting remaining fiber (Chen et al., 2019). With the increasing participation of fermentation, we found that the bacterial community in the hindgut was more easily affected than in the small intestine.

While our attention has been focused on the hindgut, some changes in microflora were observed. Family Ruminococcaceae was the most abundant family, genus *Ruminococcacea_unidentified* was the most abundant genus. Family Ruminococcaceae is a cellulolytic bacteria, which converts cellulose and sugars to acetate, propionate, and succinate (Liu et al., 2018). A recent study showed that inflammatory bowel diseases (IBD) could cause disturbances in an association network of the families Lachnospiraceae and Ruminococcaceae (Yilmaz et al., 2019). In our study, an increase of the relative abundance of the family Ruminococcaceae occurred with a decrease of the family Lachnospiraceae in HRDS in the cecum. Combined with butyrogenic capability of the family Lachnospiraceae (Zhang et al., 2019), we presumed that intestinal inflammation caused by a high RDS level diet might induce an imbalance between Lachnospiraceae and Ruminococcaceae in the hindgut. This intestinal flora disturbance resulted in a decrease of butyrate: acetate (B: A) ratio in HRDS. We also found an increase of relative abundance of the families Succinivibrionaceae and *Succinivibrio* accompanied by elevated concentration of propionate molar proportion in the MRDS group at the rectum. Succinivibrionaceae and *Succinivibrio* have been proven to be positively correlated with the propionate concentration in rumen, the one reason is that the genus *Succinivibrio* could promote the digestion of cellulose and hemicellulose, another reason is succinate can be further decarboxylated to form propionate (Pope et al., 2011; Sun et al., 2016; Ren et al., 2019). Previous study found that the concentration of propionate in the hindgut was higher in pigs fed with a high amylose starch diet (Gao et al., 2020). Considering Succinivibrionaceae and *Succinivibrio* were both positively correlated with the molar proportion of propionate, we speculated that the MRDS group with appropriate amounts of RDS in the hindgut could promote the synthesis of propionate. In addition, as butyrate-producing bacteria, *Bacteroidales_S24-7* and *Bacteroidales_S24-7_group_unidentified* also alerted in the hindgut with an increased relative abundance in LRDS. Genome analysis revealed the *Bacteroidale_S24-7_spp.* is versatile with complex carbohydrate degradation and butyrate production (Lagkouvardos et al., 2019). Our findings supported this positive correlation between the *Bacterfamilyoidale_S24-7 group* (family Bacteroidale_S24-7) and butyrate in the hindgut. We hypothesized that the butyrate molar proportion was increased due to the high abundance of the *Bacterfamilyoidale_S24-7 group* (family Bacteroidale_S24-7) and high amylose in the LRDS group.

The microbial communities in the cecum were predicted with BugBase phenotypic prediction. We observed that the LRDS group had low cecal potentially pathogenic microflora abundance and strong oxidative stress tolerability compared with the HRDS group at the cecum. Against colonization of potentially harmful microorganisms in the intestine is a principal function for bacteria to protect the host (Rivera-Chavez et al., 2016). Some anaerobes frequently suffered from oxidative stress because anaerobic metabolic pathways were inhibited (Fu et al., 2015). Based on microbial community phenotypes, we concluded that LRDS is helpful for preventing the colonization of potentially pathogenic phenotypes and creating an environment for anaerobes in the hindgut.

Metagenomic approaches based on high-throughput sequencing have rapidly facilitated the study of intestinal microbiota in livestock right behind mice (Liu et al., 2019; Xue et al., 2020). Based on metagenomics, sequencing results showed a high abundance of specie *Succinatimonas_sp._CAG:777* in MRDS group. In our study it was the second most abundant cecal specie belonging to the lineage of Proteobacteria (phylum), Gammaproteobacteria (class), Aeromonadales (order), Succinivibrionaceae (family), and *Succinatimonas* (genus), but the investigations on these bacteria in ruminants are very poor (Qi et al., 2019). Our study first found *Succinatimonas_sp._CAG:777* present in ruminants’ hindguts. It was the predominantly affected species and proved the positive correlation between the molar proportion of propionate and genus *Succinatimonas* in the cecum. Considering succinate can be further decarboxylated to the form of propionate by Wood–Werkman cycle in propionate-producing bacteria (Wood and Werkman, 1938), we suggested that this study may provide a new direction to study the relationship between dietary carbohydrate and intestinal SCFAs-producing strain in ruminants. Similar to previous studies in goats, we found Firmicutes was the dominant phylum in the cecum (Jin et al., 2018). We observed an increase of genus *Mycoplasma* in the HRDS groups. The study showed that as fermentation patterns of cellulolytic fungi, *Mycoplasma* could produce acetate as major metabolites (Rimoldi et al., 2019). However, as no members of *Mycoplasma* could produce digestive enzymes without symbiotic bacteria (Conche and Greten, 2018), a further study of intestinal symbiosis bacteria is undoubtedly required.

Functional capacities were performed by metagenomic analysis. CAZymes-encoding gene abundance and KEGG enzyme gene abundance were helpful to characterize carbohydrate degradation for dairy goats (Stewart et al., 2018). CAZymes metagenomic analysis has shown that glycoside hydrolase (GH) families were the most abundant in the cecum, which is consistent with our published result in the rumen (Shen et al., 2020b). Non-starch polysaccharides including cellulose are the main available energy source for microbes in the cecum (Gray, 1947). During degradation, the cellulose fibrils attacked by exoglucanase (EC3.2.1.91) are generally from GH families 6, 7, and 48. The endoglucanases (EC 3.2.1.4), which cleaved the cellulose chain internally, are generally from GH families 5, 6, 7, 9, 10, and 45. The β-1,4-glucosidase(EC 3.2.1.21), which hydrolyzed the non-reducing β–D-glucose bond, are generally from GH families 1, 3, 5, and 9 (Cairo et al., 2013; Morais and Mizrahi, 2019). In our study, gene-encoded cellulases mainly belonged to GH9, GH10, GH5, and GH88 families. The second most abundant family GH10 has β-1,4-glucanase activity (Zhao et al., 2019). Adding recombinant endoglucanase of GH10 can increase the digestibility of DM, NDF, and ADF *in vitro* ruminal culture (Ribeiro et al., 2018). We found that the abundance of the GH10 family was higher under HRDS treatment. Consistent with early research in rumen, we concluded that the HRDS diet could decrease cellulase activity by down-regulating endoglucanases in rumen, and the un-degraded cellulose which flowed into the hindgut could lead to an increasement of cecal cellulose content.

In addition, we found that LRDS and MRDS diets significantly increased gene abundance of GH13_11 and GH13_20. A previous study has shown that GH13_11 (isoamylase and glycogen debranching enzymes) and GH13_20 (pullulanase and pullulan hydrolase) belong to GH13 subfamilies as amylopectin degradation enzymes (Møller et al., 2016). Isoamylase could specifically attack α-1,6-glucosidic linkages at the branch to leave amylose (Li et al., 2017). We found a high concentration of amylose which was consistent with the high isoamylase gene abundance in the LRDS group. Combined with our previous study in rumen, we believed the decreased starch branching enzymes could cause some of the starch which escapes from the rumen to be passed into the hindgut in the LRDS group (Shen et al., 2020b). We speculated that the extensive amylose made efficient microorganisms in the hindgut for amylopectin degradation.

Using PROBIO database to help characterize the contribution of host probiotics is a new trend to improve gut health (Tao L. et al., 2017). In the present study, a total of 128 probiotics were detected and seven changed noticeably with the same trend, which was decreased in the HRDS group compared with the LRDS and MRDS groups. We found five probiotics related to intestinal anti-inflammation which were decreased in the HRDS group. A previous study has shown that *Bacillus subtilis* and *Bacillus Coagulans* could improve abdominal pain and diarrhea in irritable bowel syndrome (IBS) patients (Rogha et al., 2014). *Lactobacillus equigenerosi* and *Lactobacillus paracasei* belonging to the genus *Lactobacillus* could activate macrophages to suppress inflammation in IBS patients (Koryszewska-Baginska et al., 2019). *Lactococcus lactis* ssp. *Cremoris* could limit tissue injury in the intestine (Naudin et al., 2020). In our study, we found that goats fed the HRDS diet had higher free LPS concentrations in plasma than under LRDS or MRDS diets. We also found a significant correlation between the dietary RDS content and plasma LPS concentration (Shen et al., 2020a). Based on the above studies, we speculated that a lack of anti-inflammatory probiotics might cause intestinal tissue damage and inflammation in HRDS, and the increased free LPS in the circulatory system may be responsible.

In addition to microbiota dysbiosis, inflammatory response was another important consequence of a high-concentrate diet for goats (Tao S. Y. et al., 2017). In our previous study, we observed lipopolysaccharide (LPS) concentration increased in the jugular vein of the HRDS group (Shen et al., 2020a). The increase of circulating LPS is mainly absorbed from the large intestine rather than the rumen (Chang et al., 2019). We measured mRNA level of inflammatory markers and observed the expression of the cytokine IL-1β and SIgA were upregulated in the cecal mucosa of the HRDS group. A previous studies has shown inflammation could be promoted by IL-1β and stimulated the expression SIgA against the adhesion of pathogens in colonic inflammation (Liu et al., 2014; Tao S. Y. et al., 2017). We found that the expression of Muc2 in the cecal epithelium was decreased by immunohistochemistry, which could protect the intestinal epithelial cells from spontaneous colitis (Johansson et al., 2014). In this study, the phenotypes such as decreased probiotics, increased butyrate-producing bacteria, and expression of IL-1β were identically observed in mice with deleted Muc2 (Johansson et al., 2008). Overall, we speculated that feeding a HRDS diet to dairy goats could cause hindgut mucosal injuries *via* epithelial damage and inflammation.

## Conclusion

In conclusion, this study investigated changes in intestinal fermentation, composition of bacterial in the hindgut, and CAZymes-encoding gene and KEGG enzyme gene related to fiber and starch degradation in the cecum in dairy goats with different dietary RDS. We revealed that dietary RDS altered intestinal bacterial community and carbohydrate digestibility in dairy goats. The HRDS group increased the gene abundance of cellulase enzyme, and then acetate proportion in the cecum. The LRDS group increased the butyrate molar proportion by increasing the abundance of butyrate producer bacterial families and gene abundance of probiotics, and then increased the expression of Muc2 *SIgA* in cecal mucosa at the cecum. The MRDS group increased the propionate proportion by increased succinate-producing bacteria abundance. The HRDS group decreased amylose content and increased cellulose content in the cecum. Therefore, this study can enhance our understanding of hindgut starch and cellulose degradation, and cecal epithelial inflammation in dairy goats with the change of dietary RDS level.

## Data Availability Statement

The datasets presented in this study can be found in online repositories. The names of the repository/repositories and accession number(s) can be found below: https://www.ncbi.nlm. nih.gov/, PRJNA706869.

## Ethics Statement

The animal study was reviewed and approved by Institutional Animal Care and Use Committee of Northwest A&F University.

## Author Contributions

XH, JS, LZ, and JY designed the research. XH, XY, JS, LZ, and CJ performed the experiment. XH, XL, and XY analyzed the data and wrote the manuscript. XL undertook revision work. JY, YC, and XL finalized the manuscript. All authors read and approved the final version of the manuscript.

## Conflict of Interest

The authors declare that the research was conducted in the absence of any commercial or financial relationships that could be construed as a potential conflict of interest.

## Footnotes

1 http://drive5.com/uparse/

2 https://bugbase.cs.umn.edu/index.html

3 www.illumina.com

4 https://github.com/voutcn/megahit

5 http://www.bioinformatics.org/cd-hit/

6 https://github.com/weizhongli/cdhit

7 http://www.hmmer.org/

8 www.majorbio.com

## Supplementary Material

Supplementary Figure 1 Effect of dietary RDS on the content of cellulose and amylose in the cecum digesta.

Supplementary Figure 2 A significant number of bacterial based on 16s rRNA sequence dataset between LRDS, MRDS, and HRDS groups.
