# Supplementary material for: A Metagenomic Insight Into the Hindgut Microbiota and Their Metabolites for Dairy Goats Fed Different Rumen Degradable Starch
Source: Front Microbiol. 2021 Jun 7;12:651631. doi: 10.3389/fmicb.2021.651631 (PMC8216219; doi:10.3389/fmicb.2021.651631)
Supplement: Supplementary file 1 [file Data_Sheet_1.docx]

**Table S1 The composition and ingredients of experimental diets.**

| Item | Treatments | | |
| --- | --- | --- | --- |
|  | LRDS | MRDS | HRDS |
| Ingredient, (% of DM) |  |  |  |
| Alfalfa hay | 17.50 | 17.50 | 17.50 |
| Corn silage | 27.50 | 27.50 | 27.50 |
| Corn | 40.00 | 23.50 | 8.00 |
| Wheat | - | 18.00 | 36.00 |
| Soybean meal | 13.00 | 7.60 | 5.00 |
| Corn gluten meal | - | 1.90 | 2.00 |
| Wheat bran | - | 2.00 | 2.00 |
| Calcium phosphate | 0.25 | 0.25 | 0.25 |
| Limestone | 0.75 | 0.75 | 0.75 |
| Salt | 0.50 | 0.50 | 0.50 |
| Vitamin-mineral mix^1^ | 0.50 | 0.50 | 0.50 |
| Nutrient composition, (% of DM) unless noted | |  |  |
| DM | 50.58 | 50.27 | 50.21 |
| ADF | 18.88 | 18.58 | 18.22 |
| NDF | 34.38 | 33.76 | 32.90 |
| CP | 16.40 | 16.71 | 16.59 |
| Starch | 27.66 | 27.54 | 28.58 |
| RDS | 20.52 | 22.15 | 24.88 |
| NE_L_^2^，MJ/kg | 7.14 | 7.11 | 7.11 |

^1^ Vitamin-mineral mix (per kilogram): 450 mg of nicotinic acid, 600 mg of Mn, 950 mg of Zn, 430 mg of Fe, 650mg of Cu, 30 mg of Se, 45 mg of I, 20 mg of Co, 800 mg of vitamin E, 45,000 IU of vitamin D, and 120,000IU of vitamin A.

^2^ RDS: rumen degradable starch, percentage of total starch. Assuming a rumen outflow rate of 6%/h.

**Table S2** **Microbial relative abundance based on NR database significantly changed among groups in cecum.**

| Item | Treatments | | | | | | *P* value |
| --- | --- | --- | --- | --- | --- | --- | --- |
|  | LRDS  -mean | SD | MRDS  -mean | SD | HRDS  -mean | SD |  |
| s__Succinatimonas_sp._CAG:777 | 2.811 | 1.738 | 3.587 | 5.435 | 0.292 | 0.344 | 0.033 |
| g__unclassified_p__Firmicutes | 9.402 | 1.312 | 7.443 | 1.308 | 9.887 | 1.052 | 0.029 |
| g__unclassified_f__Lachnospiraceae | 3.753 | 0.828 | 3.670 | 0.785 | 2.759 | 0.416 | 0.044 |
| g__Ruminococcus | 2.785 | 0.457 | 3.157 | 0.765 | 4.004 | 0.491 | 0.007 |
| g__Succinatimonas | 2.909 | 1.799 | 3.746 | 5.669 | 0.303 | 0.349 | 0.033 |
| g__Oscillibacter | 1.433 | 0.310 | 1.097 | 0.189 | 1.411 | 0.098 | 0.036 |
| f__unclassified_p__Firmicutes | 9.402 | 1.312 | 7.443 | 1.308 | 9.887 | 1.052 | 0.029 |
| f__Ruminococcaceae | 6.619 | 1.048 | 6.835 | 1.797 | 8.813 | 0.873 | 0.012 |
| f__Succinivibrionaceae | 4.161 | 2.876 | 5.048 | 7.544 | 0.407 | 0.400 | 0.046 |
| f__Oscillospiraceae | 1.509 | 0.327 | 1.151 | 0.199 | 1.474 | 0.103 | 0.039 |
| o__unclassified_p__Firmicutes | 9.402 | 1.312 | 7.443 | 1.308 | 9.887 | 1.052 | 0.029 |
| o__Aeromonadales | 4.197 | 2.925 | 5.060 | 7.552 | 0.416 | 0.398 | 0.048 |
| o__Mycoplasmatales | 0.676 | 0.305 | 0.187 | 0.039 | 0.403 | 0.360 | 0.017 |
| c__unclassified_p__Firmicutes | 9.402 | 1.312 | 7.443 | 1.308 | 9.887 | 1.052 | 0.029 |
| c__Mollicutes | 0.892 | 0.357 | 0.361 | 0.082 | 0.595 | 0.346 | 0.022 |
| p__Tenericutes | 0.595 | 0.346 | 0.892 | 0.357 | 0.361 | 0.082 | 0.022 |

**Table S3 Comparisons of** **the gene abundance of the KEGG pathway in carbohydrate metabolism.**

| Item | Treatments | | | | | | *P* value | Corrected  *P* value |
| --- | --- | --- | --- | --- | --- | --- | --- | --- |
|  | LRDS  -mean | SD | MRDS  -mean | SD | HRDS  -mean | SD |  |  |
| Starch and sucrose metabolism | 29.490 | 1.159 | 31.430 | 2.541 | 27.470 | 1.306 | 0.022 | 0.045 |
| Glycolysis / Gluconeogenesis | 27.770 | 0.528 | 26.860 | 1.154 | 28.880 | 0.494 | 0.006 | 0.031 |
| Amino sugar and nucleotide sugar metabolism | 12.450 | 0.290 | 12.190 | 0.346 | 12.510 | 0.197 | 0.280 | 0.382 |
| Pyruvate metabolism | 25.220 | 0.821 | 24.380 | 1.243 | 26.160 | 0.633 | 0.038 | 0.045 |
| Galactose metabolism | 8.635 | 0.316 | 8.574 | 0.520 | 8.732 | 0.273 | 0.782 | 0.838 |
| Pentose phosphate pathway | 6.829 | 0.142 | 6.709 | 0.210 | 6.662 | 0.112 | 0.150 | 0.309 |
| Glyoxylate and dicarboxylate metabolism | 6.750 | 0.224 | 6.528 | 0.323 | 6.863 | 0.217 | 0.219 | 0.350 |
| Citrate cycle (TCA cycle) | 14.000 | 0.534 | 13.550 | 0.719 | 14.520 | 0.307 | 0.045 | 0.045 |
| Butanoate metabolism | 6.012 | 0.243 | 6.025 | 0.207 | 6.092 | 0.145 | 0.742 | 0.838 |
| Fructose and mannose metabolism | 5.612 | 0.176 | 5.398 | 0.410 | 5.726 | 0.140 | 0.233 | 0.350 |
| Propanoate metabolism | 4.604 | 0.188 | 4.650 | 0.461 | 4.603 | 0.114 | 0.977 | 0.977 |
| Pentose and glucuronate interconversions | 3.091 | 0.168 | 2.958 | 0.400 | 3.286 | 0.164 | 0.132 | 0.309 |
| C5-Branched dibasic acid metabolism | 3.525 | 0.447 | 3.781 | 0.725 | 2.970 | 0.192 | 0.033 | 0.045 |
| Ascorbate and aldarate metabolism | 0.694 | 0.058 | 0.664 | 0.056 | 0.672 | 0.059 | 0.686 | 0.838 |
| Inositol phosphate metabolism | 0.631 | 0.047 | 0.602 | 0.063 | 0.715 | 0.037 | 0.009 | 0.118 |
