# Supplementary figures and images for: A Metagenomic Insight Into the Hindgut Microbiota and Their Metabolites for Dairy Goats Fed Different Rumen Degradable Starch
Source: Front Microbiol. 2021 Jun 7;12:651631. doi: 10.3389/fmicb.2021.651631 (PMC8216219; doi:10.3389/fmicb.2021.651631)

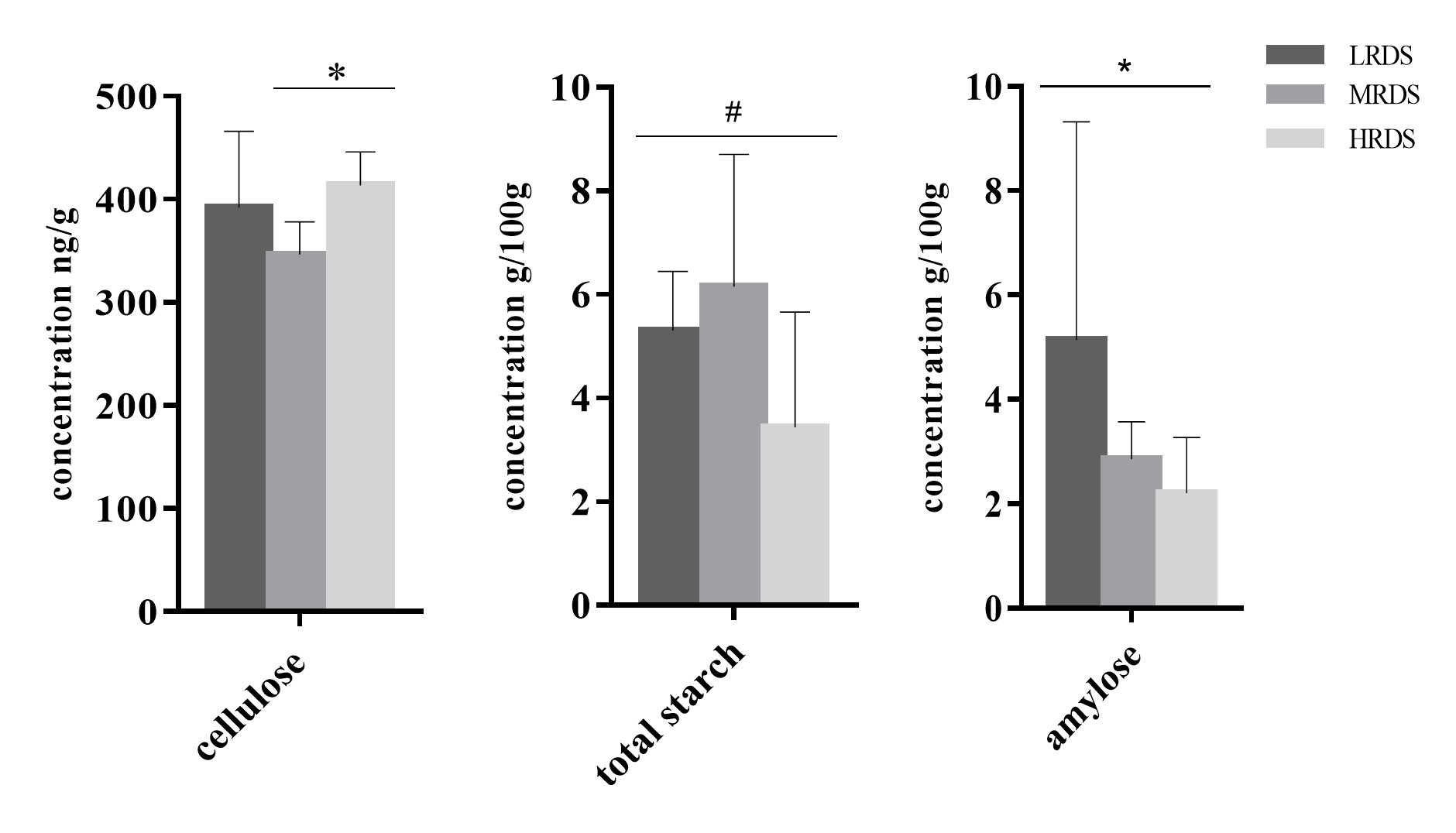

Supplement: Supplementary Figure 1 — Effect of dietary RDS on the content of cellulose and amylose in the cecum digesta. [file Image_1.JPEG]

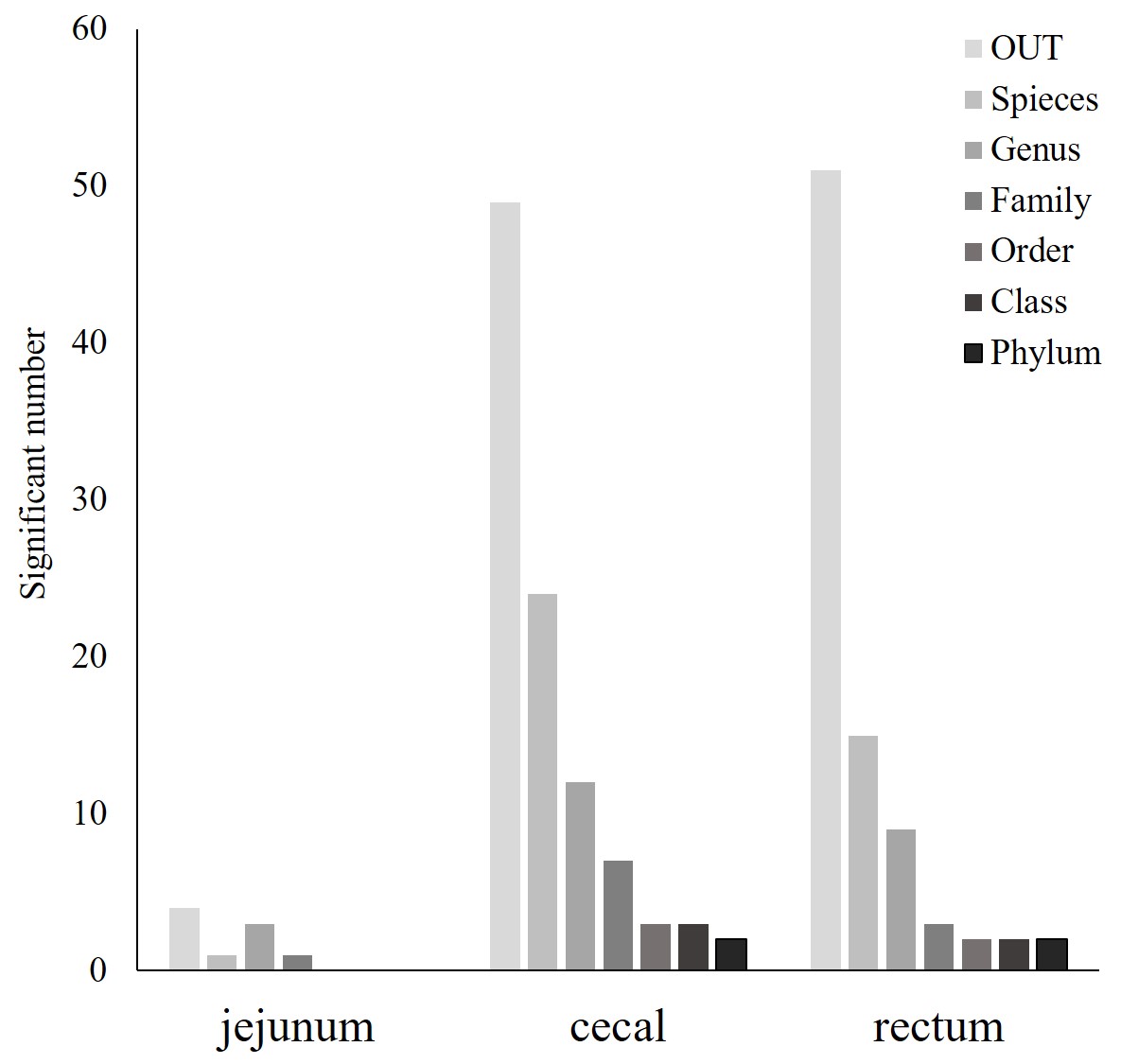

Supplement: Supplementary Figure 2 — A significant number of bacterial based on 16s rRNA sequence dataset between LRDS, MRDS, and HRDS groups. [file Image_2.JPEG]
